# Shedding Light on the Cell Biology of the Predatory Bacterium *Bdellovibrio bacteriovorus*

**DOI:** 10.3389/fmicb.2019.03136

**Published:** 2020-01-21

**Authors:** Géraldine Laloux

**Affiliations:** de Duve Institute, Université catholique de Louvain, Brussels, Belgium

**Keywords:** *Bdellovibrio bacteriovorus*, cell cycle, bacterial cell biology, predation, chromosome dynamics, microscopy, polarity, cell division

## Abstract

*Bdellovibrio bacteriovorus* is a predatory bacterium that feeds upon and proliferates inside other Gram-negative bacteria. Upon entry into the periplasmic space of the prey envelope, *B. bacteriovorus* initiates an exquisite developmental program in which it digests the host resources and grows as a filament, which eventually divides in a non-binary manner, releasing a variable number of daughter cells. The progeny then escape from the prey ghost to encounter new victims and resume the predation cycle. Owing to its unique biology, *B. bacteriovorus* undoubtedly represents an attractive model to unravel novel mechanisms of bacterial cell cycle control and cellular organization. Yet, the molecular factors behind the sophisticated lifestyle of this micro-predator are still mysterious. In particular, the spatiotemporal dynamics of proteins that control key cellular processes such as transmission of the genetic information, cell growth and division remain largely unexplored. In this Perspective article, I highlight outstanding fundamental questions related to these aspects and arising from the original biology of this bacterium. I also discuss available insights and potential cell biology approaches based on quantitative live imaging techniques, in combination with bacterial genetics and biochemistry, to shed light on the intracellular organization of *B. bacteriovorus* in space and time.

## Specific Spatiotemporal Patterns Inside Bacteria Underlie Their Sophisticated Cell Cycles

Despite the general lack of intracytoplasmic organelles, bacterial cells are remarkably organized in space and time. Indeed, their content is distributed at specific subcellular locations in a sophisticated and dynamic manner. Such spatiotemporal organization allows various cellular processes to be tightly coordinated with each other, which is absolutely crucial for the proper completion of every cell cycle and for successful adaptation to the environment. The fine regulation of key cellular events (such as DNA replication, chromosome segregation, cell growth and division) by the dynamic positioning of the corresponding regulators inside the cell is the topic of intense research (Reyes-Lamothe and Sherratt, 2019). Massive textbook knowledge has been acquired during the past 20 years or so, thanks to the monitoring of reporter proteins by live fluorescence microscopy in a handful of model organisms including *Escherichia coli*, Bacillus subtilis, and *Caulobacter crescentus.* These localization studies are progressively revealing general principles of spatiotemporal organization in bacterial cells (Surovtsev and Jacobs-Wagner, 2018). Intriguingly, it often turns out that different species exploit conserved actors to control major cellular events, but fine-tune the biochemical properties, physical partners or localization dynamics of these proteins to achieve distinct molecular choreographies and thus, markedly different lifecycles. Another prevalent finding is that the cell poles constitute particular areas in which many proteins accumulate to regulate these events (Shapiro et al., 2009; Laloux and Jacobs-Wagner, 2014). Cell division intrinsically generates asymmetry in each newborn cell, as one “old” cell pole is inherited from the mother cell while one “new” pole is formed during division. Remarkably, polarity and hence, asymmetry in the cell, are critical for a variety of aspects of bacterial life, including cell cycle regulation, motility or virulence (Laloux and Jacobs-Wagner, 2014; Surovtsev and Jacobs-Wagner, 2018; Reyes-Lamothe and Sherratt, 2019). In the past years, the focus of bacterial cell biology research has expanded from the few first model species to a broader range of bacteria featuring highly diverse lifestyles (Eswara and Ramamurthi, 2017), providing suitable platforms to investigate novel physiological and molecular mechanisms.

In this Perspective article, I will first present the predatory bacterium *Bdellovibrio bacteriovorus* as a non-canonical model to address outstanding questions in the field of bacterial cell biology, due to its stunning lifestyle and proliferation mode. Then I will focus on select unresolved aspects that emerge from the biology of *B. bacteriovorus* concerning to the spatiotemporal regulation of the key cellular processes mentioned above, i.e., how these bacteria transfer their genetic information, how they grow, divide and establish cellular polarity in their multiple progeny. I will briefly summarize a few insights that have been obtained so far and emphasize how quantitative live cell imaging could contribute to a single-cell level and molecular understanding of the *B. bacteriovorus* cell cycle.

## *Bdellovibrio bacteriovorus* as a new model in bacterial cell biology: how does a non-binary bacterium organize its cell content in space and time?

**FIGURE 1:**
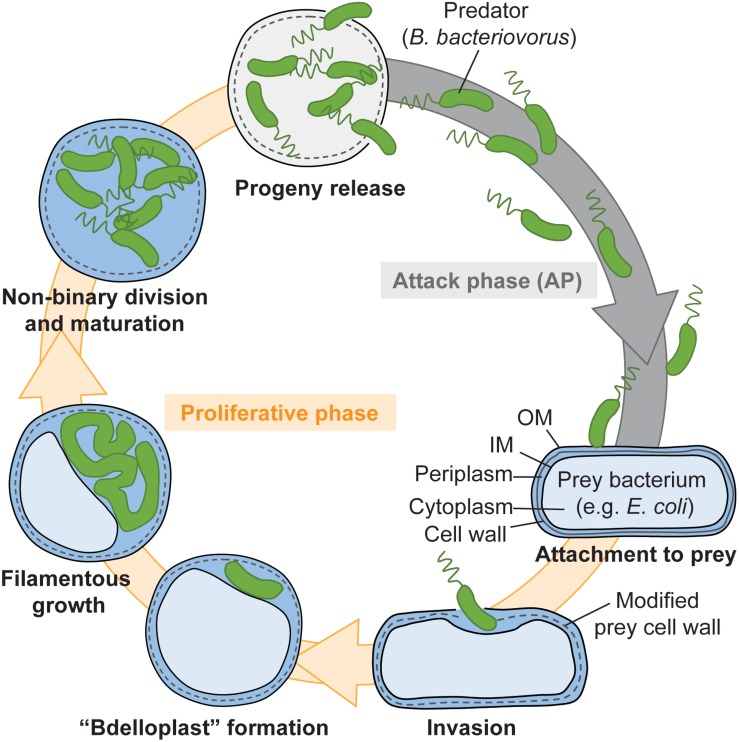
Predatory lifecycle of *Bdellovibrio bacteriovorus*. 1. Mono-flagellated *B. bacteriovorus* cells swim in the attack phase. 2. Upon contact with a prey, the predator attaches to it by the non-flagellated pole via Type IVa pili (Evans et al., 2007; Mahmoud and Koval, 2010). 3. The predator squeezes through a narrow pore in the prey outer membrane and cell wall, which appears to be resealed after predator entry (Kuru et al., 2017). 4. *B. bacteriovorus* produces an arsenal of lytic enzymes, including peptidoglycan-degrading ones, leading to a modification of the cell wall and rounding of the prey (called bdelloplast) (Lerner et al., 2012), and digestion of the prey cytosolic content. A progressive shift in the transcriptional program of *B. bacteriovorus* is triggered by prey contact and sensing of undetermined intracellular prey content, and allows it to transition from the attack phase to the growth phase (Lambert et al., 2010; Karunker et al., 2013; Rotem et al., 2015). 5. *B. bacteriovorus* grows as a filament and presumably synthesizes an odd or even number of chromosomal copies. What determines the extent of intra-periplasmic growth and the timing of progeny release is unclear. 6. The mother cell undergoes an atypical, simultaneous non-binary division (Fenton et al., 2010b). 7. Daughter cells mature into attack phase cells before escaping in the environment. The full cycle takes ∼4 h in laboratory conditions. Rare spontaneous mutants acquire the ability to proliferate in a host-independent (HI) manner (not depicted here). A first mutation, often in a gene that may directly or indirectly regulate extrusion of the Type IVa pili required for prey attachment and invasion (Evans et al., 2007; Mahmoud and Koval, 2010; Wurtzel et al., 2010; Capeness et al., 2013), allows the cells to grow outside a prey in rich medium (Cotter and Thomashow, 1992; Roschanski et al., 2011; Capeness et al., 2013). A second mutation affecting the RNA degradosome, commits *B. bacteriovorus* to an irreversible HI life, in which it loses predation ability (Roschanski et al., 2011). I refer the reader to excellent publications for in-depth review of the *B. bacteriovorus* lifestyle (Sockett, 2009; Rotem et al., 2014; Negus et al., 2017).

Back in the 1960’s, researchers had spotted the original lifestyle of a vibrioid, tiny bacterium (0.3–0.5 μm wide, 0.8–1.2 μm long) that was serendipitously isolated in a quest for bacteriophages (Stolp and Petzold, 1962) and suitably named *B. bacteriovorus* (Stolp and Starr, 1963). It was later determined that this bacterium belongs to the δ-*proteobacteria* (Davidov and Jurkevitch, 2004), a group of Gram-negative species including the well-studied wolf-pack forming predator *Myxococcus xanthus*. The most extraordinary feature of *B. bacteriovorus* lifecycle [Figure 1, reviewed in Sockett (2009); Rotem et al. (2014), Negus et al. (2017)] is the fact that it uses other Gram-negative bacteria as a niche to proliferate (Starr and Baigent, 1966). A couple of spatially and temporally separated prey cues, which are proposed to involve unknown compounds of the prey envelope and cytosol, trigger a stepwise transition from the non-replicating attack-phase to the growing stage of the *B. bacteriovorus* cell cycle (Rotem et al., 2015). Following cell division inside their victim, newborn *B. bacteriovorus* are released into the wild where they swim to encounter new prey. Interestingly, rare spontaneous mutations allow *B. bacteriovorus* to bypass the need for prey to proliferate [reviewed in Thomashow and Cotter (1992); Rotem et al. (2014), see also legend of Figure 1]. However, the molecular effects of these mutations and the modes of elongation and division of these so-called host-independent (HI) mutants are still unclear.

A second prominent facet of the *B. bacteriovorus* cell cycle is the non-binary mode of growth and division that this bacterium has adopted to proliferate inside its prey. Instead of doubling its size, replicating its genomic content once and relocating it in the future daughter cells before dividing in two cells (which textbook model bacteria do), *B. bacteriovorus* elongates to reach more than twice its initial length, before dividing in a variable, odd or even number of daughter cells (Thomashow and Rittenberg, 1979; Fenton et al., 2010b). This raises exciting fundamental questions related to the spatiotemporal organization of the factors underlying growth, polarity, transmission of the genetic information and cell division – aspects that are extensively explored in model bacteria but remain mysterious in *B. bacteriovorus*. Some of these questions had already been cleverly pointed out by the researchers who led the wonderful early investigations on the biology of this curious predator (Thomashow and Rittenberg, 1979).

### Transfer of the Genetic Information

The *B. bacteriovorus* filament indifferently produces odd or even offspring (Fenton et al., 2010b), in sharp contrast with the binary division of most model bacteria. This implies that the circular chromosome from the parental cell is replicated to produce either an odd or even copy number, a problem that remains largely unexplored. One possibility to explain an equally frequent odd or even ploidy is that only one chromosome is duplicated at a time, the replication of all other copies being inhibited. Alternatively, regulation of chromosome replication could be more complex, possibly involving the simultaneous replication of several templates. The latter is supported by (1) pulse-labeling experiments with radioactive thymidine indicating multiple sites of insertion in the cell, presumably reflecting areas where DNA replication occurs (Gray and Ruby, 1989); and (2) a recent report in which several foci of a fluorescently tagged copy of the replisome β-clamp DnaN were seen in *B. bacteriovorus* growing inside *E. coli* cells (Makowski et al., 2019), suggesting that more than one replisome can be assembled at the same time. Yet, the spatiotemporal dynamics of the native replisome in the predator cell remain elusive. Moreover, the potential mechanism that controls which *ori* serves as a platform for initiating the next replication round, and whether DNA replication initiation and termination are coupled to cell growth and prey consumption, are completely unknown. Recording the position, number and time of assembly of the replisome(s), using automated image analysis of large numbers of cells expressing fluorescent components of the replication machinery at native levels, should help deciphering where and when DNA replication occurs within the predator cell as it resides inside the prey. The major regulator of DNA replication initiation DnaA is present in *B. bacteriovorus* (Makowski et al., 2016) and constitutes a possible starting point for mutational, localization and interaction analyses to complement replisome dynamics studies and obtain the first clues on how this central process is integrated with filamentous growth in the context of a limited amount of resources.

In canonical models, the newly duplicated origins of replication are pulled apart early on by the segregation machinery, well before the rest of the chromosome is copied (Reyes-Lamothe et al., 2011; Wang et al., 2013). The *B. bacteriovorus* chromosome includes typical DNA sequences and encodes homologs of the highly conserved ParABS partitioning system (Rendulic et al., 2004; Makowski et al., 2016, 2019), which is extensively characterized in other species and involved in partitioning chromosomal copies in the right direction (Wang et al., 2013). However, how chromosome segregation is achieved and coordinated with other events has never been examined in *B. bacteriovorus*. Addressing these questions may lead to the discovery of novel mechanisms of chromosome processing (i.e., from their synthesis to their segregation in future daughter cells). An important feature that contributes to orchestrating replication and segregation is the spatial arrangement of the chromosome within the cell, i.e., the relative position of key loci, which vary greatly among species (Webb et al., 1997; Li et al., 2002; Viollier et al., 2004; Espeli et al., 2008). It is likely that loci localization assays in living cells, such as the Fluorescent Reporter-Operator System (FROS) (Robinett et al., 1996; Lau et al., 2003), will not only reveal the position and dynamics of chromosomal loci in *B. bacteriovorus*, which have never been explored so far, but also provide important insights into how this bacterium deals with multiple DNA replication and segregation throughout its cell cycle.

### Predator Cell Growth

Distinct modes of cell elongation have been proposed for *B. bacteriovorus*: (1) unipolar growth (Eksztejn and Varon, 1977; Friedberg, 1978), (2) bipolar growth (Fenton et al., 2010b), (3) or dispersed growth along the cell by addition of new material in multiple patches (Gray and Ruby, 1989), as also recently suggested by Structured Illumination Microscopy imaging of fluorescent di-amino acids that label sites of peptidoglycan (PG) insertion (Kuru et al., 2017). Thus, these seemingly contrasting results ask for in-depth investigation of the exact mechanism of spatial control of PG incorporation. Intriguingly, predator cells almost double their size between birth inside the bdelloplast and the prey-free stage, as observed by electron microscopy (Fenton et al., 2010b). Further quantitative analysis of cell length should clarify when the predator cell actually grows, and to which extent. The use of living cells will be critical, not only to bring temporal insights but also to exclude potential effect of fixation on cellular morphology.

A key component of the PG synthesis machinery during elongation is the actin homolog MreB, which contributes in directing cell wall assembly and therefore constitutes a cell shape determinant (Errington, 2015). *B. bacteriovorus* harbors two copies of *mreB*, which are both essential for viability. Although the pleiotropic phenotypes of merodiploid strains carrying a fluorescent fusion of MreB1 or MreB2 suggest that they play distinct roles during intra-periplasmic development (Fenton et al., 2010c), more work is needed to understand the importance of having two copies of MreB for the lifecycle of the predator and to uncover their function in cell elongation or other unsuspected processes. Tracking the behavior, and possibly the motion of non-disruptive fluorescent fusions to MreB1 and MreB2 at several cell cycle stages, as done for the *E. coli* or *B. subtilis* MreB using epifluorescence microscopy or Total Internal Reflection Fluorescence Microscopy, respectively (Dominguez-Escobar et al., 2011; Garner et al., 2011; van Teeffelen et al., 2011), together with protein-protein interaction assays to fish potential partners, will help discovering the role(s) of these actin homologs in *B. bacteriovorus*.

### Spatiotemporal Control of Non-binary Cell Division

What determines the timing and the localization of the division events that split the predator filament into a variable number of daughter cells? This is certainly one of the most striking questions that comes to mind when observing *B. bacteriovorus* proliferating inside their prey. In model species, spatiotemporal control of cell division often invokes a negative regulator that prevents division at a given time and/or place in the cell. For instance, nucleoid occlusion factors hinder division over non-partitioned chromosomes (Wu and Errington, 2004; Bernhardt and de Boer, 2005; Mercy et al., 2019), whereas the Min system inhibits division at cell poles (Raskin and de Boer, 1997). A few proteins that positively regulate the localization of the cytokinetic protein FtsZ have also been discovered recently (Willemse et al., 2011; Treuner-Lange et al., 2013; Fleurie et al., 2014; van Raaphorst et al., 2017; Eswara et al., 2018). Interestingly, the *B. bacteriovorus* genome lacks obvious homologs of these proteins (Rendulic et al., 2004), hinting that distinct, yet undiscovered regulators of cell division must exist in this bacterium, which may be revealed by protein-protein interaction screens using divisome elements as bait. Although several clues were obtained on the cell division process of the predator (some of which are summarized below), the nature, molecular mechanism, and regulation of the hypothetical signals controlling cell division are essentially unidentified in *B. bacteriovorus*.

Whereas some early studies postulated that the predator cell was dividing sequentially [(Burnham et al., 1970) and references therein], Fenton et al. later reported that the *B. bacteriovorus* filament divides simultaneously inside its prey (Fenton et al., 2010b). Interestingly, the progeny size (i.e., the number of daughter cells) does not seem to affect the division time (Fenton et al., 2010b). Moreover, during a coinfection, where a single prey was invaded simultaneously by two predators, the “twin” *B. bacteriovorus* filaments divided at the same time even though their progeny size was not necessarily identical, suggesting that division is elicited by nutrient deprivation or an unknown prey factor freely diffusing in the bdelloplast (Fenton et al., 2010b). Such signals could be detected by both predator cells simultaneously. Yet, the timing of division, which was estimated in this study from a visible displacement of daughter cells inside the bdelloplast, was highly variable among the <50 bdelloplasts observed (Fenton et al., 2010b). Thus, the intriguing temporal control of cell division suggested by this seminal work awaits further examination, which will require a finer assessment of constriction and division events.

Consistent with the idea that division is induced by the depletion of a prey compound, it was postulated long ago that there is a correlation between prey size and the ultimate length of the predator filament, as observed from images of fixed cells (Kessel and Shilo, 1976). This led to the generally assumed notion that the extent of growth and the timing of division are dictated by nutrient availability. Work using host-independent (HI) mutants of *B. bacteriovorus* or an artificial host-free system (Horowitz et al., 1974; Friedberg, 1978) also supported the hypothesis that cell division is triggered by depletion of a prey factor [e.g., (Varon et al., 1976)] independently of its nutritive value (Gray and Ruby, 1990), or, in contrast, by accumulation of a soluble compound released by the growing *B. bacteriovorus* (Eksztejn and Varon, 1977). Nevertheless, whether these observations fully reflect how wild-type, host-dependent cells divide in their prey is unclear. Addressing the problem of growth and division control will require single-cell level analysis of predator length and prey cell size and content over time. Readily quantifiable fluorescent reporters may constitute useful tricks to measure predator growth and several parameters of the prey cell upon live imaging.

Finally, the temporal coupling of cell division with chromosome segregation remains mysterious. Strict spatiotemporal coordination between these two fundamental processes has been demonstrated, but mostly in binary-dividing bacteria (Reyes-Lamothe et al., 2011; Wang et al., 2013; Eswara and Ramamurthi, 2017). Thus, molecular investigation of chromosome segregation in *B. bacteriovorus* may contribute to sharpen our understanding of how cell division is regulated in time and space in this bacterium.

### Setting Cell Polarity in the Progeny

Physical separation of cellular processes (e.g., enzymatic pathways) is crucial for the physiology of all cells. Remarkably, bacteria achieve functional compartmentalization by relying on “hub” proteins that have the exceptional capacity to spontaneously localize at specific regions of the cell, where they recruit various proteins including cell cycle regulators. Such regions are often specified by the geometry of the cell, i.e., the poles and septa (which become “new” poles of daughter cells upon cell division) (Laloux and Jacobs-Wagner, 2014; Treuner-Lange and Sogaard-Andersen, 2014). In particular, chromosome anchoring by polar proteins ensures spatiotemporal organization of the genomic content in model species (Wang et al., 2013). *B. bacteriovorus* is evidently polarized. For instance, (1) the predator is propelled by a unipolar flagellum and attaches to its prey by the opposite pole (Burnham et al., 1968); (2) the intermembrane distance in the envelope is wider at the invasive pole (40 nm) than along the rest of the cell (25 nm), and the flagellated pole appears rounder than the invasive pole in cryo-electron tomography (Borgnia et al., 2008); (3) RomR and an interacting protein, which are important for survival and predation but whose exact roles are unclear, localize at the invasive pole (Milner et al., 2014). Thus, several aspects of *B. bacteriovorus* lifecycle probably rely on intracellular asymmetry established by polar hubs. Yet, a fascinating implication of the non-binary mode of proliferation is that some daughter cells do not inherit an “old” pole from the mother cell, but instead only acquire “new” poles, raising the question of how and when polarity is set in the progeny. This calls once more for the analysis of protein dynamics within the growing and dividing predator. Famous polar hubs include PopZ [in alpha-*proteobacteria* (Bowman et al., 2008; Ebersbach et al., 2008)], HubP [in Vibrionaceae (Yamaichi et al., 2012)], DivIVA [in *Bacillus, Streptomyces* and other Gram-positive species (Perry and Edwards, 2004; Hempel et al., 2008; Hammond et al., 2019)] and bactofilin [a highly conserved filament-forming protein (Kühn et al., 2010)]. Interestingly, *B. bacteriovorus* genome encodes both a DivIVA (Lambert et al., 2010; Sieger and Bramkamp, 2015) and a bactofilin homolog, which therefore constitute interesting candidates for proteins involved in establishing or maintaining polarity in *B. bacteriovorus*. Investigating their subcellular distribution and identifying their potential clients may shed light on their role in the predator cell and perhaps more broadly on how polarity is achieved in non-binary dividing bacteria.

## Conclusion

How the intracellular content of *B. bacteriovorus* is distributed to achieve its original cell cycle is largely unknown. Yet, unraveling the spatiotemporal organization of these predator cells is important in at least two aspects. First, due to the exquisite proliferation mode of this species, biological questions that were not accessible with canonical model organisms can be tackled, which may illuminate novel mechanistic principles, for instance regarding the determinants of non-binary division. Second, whereas evidence accumulates supporting the use of *B. bacteriovorus* as a potential ally against pathogenic bacteria [e.g., (Atterbury et al., 2011; Shanks et al., 2013; Tyson and Sockett, 2016; Willis et al., 2016; Im et al., 2017; McNeely et al., 2017; Findlay et al., 2019; Raghunathan et al., 2019)], there is no doubt that a fundamental understanding of its biology and the underlying molecular dynamics are absolutely needed before concretely envisioning therapeutic applications. However, only a few studies aimed at visualizing proteins or other intracellular components of the predator cell inside or outside its periplasmic niche so far (Fenton et al., 2010a, b, c; Milner et al., 2014; Lambert et al., 2015; Kuru et al., 2017; Makowski et al., 2019). This can be explained by the fact that genetic tools are still relatively limited compared to the arsenal now available for model species like *E. coli*, and by the relatively small size of *B. bacteriovorus* (≤1 μm in attack phase) and its convoluted growth inside the host, which present challenges for cell biology approaches. Although highly inspiring and offering first insights into the complex lifestyle of *B. bacteriovorus*, imaging in those prior studies was either performed on fixed cells, or live imaging suffered from weak signal intensity or resolution and lacked automated quantification from high cell numbers. Future investigation based on high-end epifluorescence microscopy followed by quantitative analysis will certainly illuminate *Bdellovibrio* cell biology further. Software packages have been developed to automatically detect bacterial cells with subpixel resolution from phase contrast images, and allow quantitative assessment of protein localization over time, both at the population and single-cell levels [e.g., (Ducret et al., 2016; Paintdakhi et al., 2016)]. While these invaluable tools have been largely adopted in bacterial cell biology research, yielding unprecedented mechanistic insights from live imaging, they have never been applied to investigating intracellular dynamics of live *B. bacteriovorus*. In addition, the implementation of tailor-made image analysis tools will likely help tackling the challenges associated with intra-periplasmic growth. Conveniently, the cell cycle of *B. bacteriovorus* can be easily synchronized by adjusting the prey/predator ratio (Abram et al., 1974; Thomashow and Rittenberg, 1978), making it amenable to time-lapse analysis of cell cycle progression in large numbers of cells. Higher- or super-resolution microscopy may subsequently complement epifluorescence data to depict with finer details the subcellular patterning of the players that govern cellular processes in *Bdellovibrio*. Finally, the HI mutants of *B. bacteriovorus* may constitute a convenient approach to circumvent the challenges of intra-periplasmic imaging, offering an attractive option to facilitate the examination of intracellular patterns. A prerequisite would be to validate the proposed similarity between the host-independent and wild-type cell cycle by live microscopy, since HI strains were described as morphologically heterogenous (Seidler and Starr, 1969; Barel and Jurkevitch, 2001) and a quantified single-cell level assessment of HI growth and division outside prey is lacking. Moreover, HI mutants have unique phenotypes and transcriptome profiles (Lambert et al., 2010; Capeness et al., 2013; Rotem et al., 2014), supporting the idea that they represent a natural form of *B. bacteriovorus* (Sockett, 2009) but calling for particular caution in the interpretation of future HI-based cell biology experiments.

*Bdellovibrio bacteriovorus* is an exciting, genetically tractable tool to address biological questions that escaped the research focus in model species. It is now an empowering time to expand the field of bacterial cell biology and better grasp the diversity of the bacterial world by examining unusual microbes (Eswara and Ramamurthi, 2017). I envision that the alternative solutions deployed by *B. bacteriovorus* to ensure the transfer of genetic information, cell growth, division and polarity will reveal novel regulators or mechanisms, which could in turn enlighten how these activities are achieved in other bacteria.

## Data Availability Statement

The raw data supporting the conclusions of this article will be made available by the authors, without undue reservation, to any qualified researcher.

## Author Contributions

GL conducted literature search and review and wrote the manuscript.

## Conflict of Interest

The author declares that the research was conducted in the absence of any commercial or financial relationships that could be construed as a potential conflict of interest.
